# Clinical Spectrum of Propionic Acidaemia

**DOI:** 10.1155/2013/975964

**Published:** 2013-10-31

**Authors:** Muhammad Rafique

**Affiliations:** Department of Child Health, College of Medicine, King Khalid University, Abha 61321, Saudi Arabia

## Abstract

*Objectives*. To evaluate the clinical features, physical findings, diagnosis, and laboratory parameters of the patients with propionic acidaemia (PA). *Methods*. The records of diagnosed cases of propionic acidaemia were reviewed, retrospectively. *Results*. Twenty-six patients with PA had 133 admissions. The majority (85%) of the patients exhibited clinical manifestations in the 1st week of life. Regarding clinical features, lethargy, fever, poor feeding, vomiting, dehydration, muscular hypotonia, respiratory symptoms, encephalopathy, disturbance of tone and reflexes, and malnutrition were observed in 51–92% admissions. Metabolic crises, respiratory diseases, hyperammonaemia, metabolic acidosis, hypoalbuminaemia, and hypocalcaemia were observed in 30–96% admissions. Pancytopenia, ketonuria, hypoproteinemia, hypoglycaemia, and mildly disturbed liver enzymes were found in 12–41% admissions. Generalised brain oedema was detected in 17% and cerebral atrophy in 25% admissions. Gender-wise odd ratio analysis showed value of 1.9 for lethargy, 1.99 for respiratory diseases, 0.55 for anaemia, and 1.82 for hypocalcaemia. *Conclusion*. Propionic acidaemia usually presents with wide spectrum of clinical features and disturbances of laboratory parameters in early neonatal age. It is associated with significant complications which deteriorate the patients' quality of life. Perhaps with early diagnosis of the disease and in time intervention, these may be preventable.

## 1. Introduction

Propionic acidaemia (PA) is a rare autosomal recessive metabolic disease. About 80% are early onset cases (who are diagnosed within three months of age) which classically present in the neonatal period with lethargy, vomiting, refusal to feed, hypotonia, and less frequently with dehydration and seizures [1, 2]. Some patients show milder symptoms and long survival rate, associated with chronic late onset form [3]. In the individuals with PA, serious health problems can be triggered by prolonged fasting, fever or infections, and high protein diet leading to accumulation of toxic substances [2].

Hyperammonaemia is the most common presentation found in 88%, patients [2]. The disease is also characterized by repeated episodes of metabolic acidosis, occasionally seizures, coma, and cerebellar haemorrhages [4]. Hypoglycaemia is a commonly described finding during metabolic decompensations but rarely hyperglycaemia and decreased bone density have also been reported [5]. Commonly observed viral infection and bone marrow suppression with neutropenia and thrombocytopenia in patients with PA might be lethal [3, 6]. 

There are few reports [8] of presentation of patients with PA from Kingdom of Saudi Arabia (KSA), yet none is available from south-west region of KSA. So, this study was carried out to find out the clinical features, physical findings, laboratory parameters, and diagnosis of the diseases with which the patients with PA were admitted to the hospital. 

## 2. Methods

The study was conducted in a tertiary care, referral, public hospital, during the period of January 2001–December 2012, retrospectively. The records were ascertained of all admitted Saudi patients in the department of Paediatrics with confirmed diagnosis of PA (on basis of high propionylcarnitine level in blood, detected by tandem mass spectrometry, high level of propionate in urine, enzyme analysis, and genetic studies). All cases were reviewed for the variables of: age of the patients at diagnosis and present age, family history, clinical features, and detail of admissions. Weight and height/length of the patients were noted and their percentages of normal for age of Saudi children were calculated. Under nutrition/wasting (weight < normal for age) percentage was calculated and categorised according to Gomez classification of malnutrition. Short stature/stunting (height < normal for age) percentage was calculated and categorised according to Waterlow classification of malnutrition [10]. The records were also reviewed for biochemical analysis, other relevant investigations, and diagnosis of the presenting diseases with which those patients of PA were admitted. The data were analyzed by using SPSS version 17. One sample *t*-test (Table 2) was applied to find out the significance of important variables which produced significant impact on patients of PA. The study was approved by the Research Ethical Committee of King Khalid University, Abha, KSA (Record no. 2012-10-08).

## 3. Results

Our study group included 26 patients with 133 admissions. Their (mean ± SD) age at last admission was observed to be 4.3 ± 4.7 years.  Table 1 demonstrates demographic characteristics of patients with PA.

Regarding diagnosis of PA, tandem mass spectrometry results showed mean propionylcarnitine level of 18.8 ± 2.7 umol/L (normal: <4.33) and mean C3/C2 ratio was 1.9 ± 0.2 (normal: <0.1). In early onset cases, mean age was 2.95 ± 4.95 yr (range: 3 days–14 yr) and mean age of surviving patients was 2.24 yr (range: 3 days–12.5 yr). Frequency of admissions/year in those cases was 5.5 ± 6.7. Mean age of two late onset cases was 1.05 yr; their hospital admissions were comparatively infrequent (2/yr) and no long-term neurological complications were detected in those patients. Mean hospital stay of children per admission was 9.2 ± 8 days (range 1–25). Patients' mean ± SD weight, expected for age, was 72.4 ± 17.1% (range: 43–98, normal: >80). Six (23.1%) patients were having 1st and 2nd degree while 8 (30.1%) children had 3rd degree, under nutrition/wasting [10]. Mean height/length, expected for age, was 90.7 ± 8.8% (range: 67–101, normal: >95). Seven (26.9%) patients had 1st degree, 8 (30.1%) had 2nd degree, and 2 (7.7%) had 3rd degree stunting/short stature [10]. Ratio of the PA patient admissions to all patient admissions in the paediatrics department was 1 : 252. Summary of clinical symptoms, physical findings, and diagnoses of the presenting diseases in patients with PA is given in Table 2. Clinical features detected were loose motions in 9.8%, constipation in 6.8%, dehydration in 92.5%, hypotension in 7.5%, shock in 12%, hypotonia in 46%, hyporeflexia in 19.6%, hypertonia in 9%, and hyperreflexia in 48.9% admissions. Regarding diagnoses of PA patients, acute watery diarrhoea was found in 9% and respiratory diseases like pneumonia, chronic lung diseases, upper respiratory tract infection, and hyperactive airway disease were seen in 30.1% admissions. Among laboratory parameters, serum blood urea nitrogen (BUN) was found to be 49.7 ± 26.3 (mean ± SD), creatinine 0.66 ± 0.5 mg/dL, sodium 139 ± 9 mEq/L, and potassium 4.1 ± 0.6 mEq/L. Among liver enzymes, AST was found to be 60.6 ± 47.4, ALT 66.8 ± 106.1, ALP 172.6 ± 63.3, and GGT 94.4 ± 92.1 IU/L. Mean erythrocyte sedimentation rate was found to be 14.3 ± 9.8 mm after the 1st hour. Among disturbances in laboratory values, we found hyperammonaemia (>80 mg/dL) in 96% and severe hyperammonaemia (>1000 mg/dL) in 4.5% admissions. Hyperuraemia (BUN > 20 mg/dL) was found in 91.7%, hypercreatinemia (>1 mg/dL) in 11.3%, hypoalbuminaemia (<3.5 g/dL) in 76.7%, hyponatraemia (<135 mEq/L) in 23.3%, and hypoklamia (<3.5 mEq/L) in 7.5% admissions. Raised liver enzymes were observed in 16.6–39.9% admissions. In haematological parameters, neutropenia (absolute neutrophil count (ANC) < 1500/mm^3^) was found in 36.1% admissions, and of them, mean ANC was observed to be 892/mm^3^. Severe neutropenia (ANC < 500/mm^3^) was encountered in 5.3% admissions. Erythrocytopenia (RBCs < 3.4 × 10^6^/mm^3^) was also detected in 17.3% admissions. In arterial blood gases values, base excess >−4 was found in 94%, acidosis (HCO_3_ < 18 mmol/L) in 77%, and alkalosis (HCO_3_ > 28/mm^3^) in 8% admissions. Ketonuria was found in 35% admissions.  Table 3 summarizes clinical features and laboratory data in patients with PA and comparison of those with other studies.  Table 4 summarizes gender-wise odd ratio analysis of clinical features and laboratory findings. We applied one sample *t*-test to important variables (Table 2). Our variables were compared with those of other studies. *P* value < 0.05 indicates significant difference in these variables.

## 4. Discussion

The incidence of PA is high in Saudi Arabia (1 : 2000–5000 live births) due to a relatively high rate of intrafamilial marriages. In this study, most (92%) of the parents were consanguineous. However consanguinity was reported as 40% in another report from KSA [11]. The difference is probably due to cultural and social impact in the studied region. 

### 4.1. Clinical Presentation

The majority (84%) of the studied patients exhibited clinical manifestations in the 1st week and 92% in the first 2 weeks of life, while these figures were reported as 66% and 80%, respectively, in an Austrian study [2]. In this study, 51–92% admissions of early onset cases especially of neonatal age presented with nonspecific symptoms like lethargy (odd ratio 1.69—Table 4), fever, poor feeding, vomiting, dehydration, muscular hypotonia, respiratory symptoms, severe acidosis, metabolic crisis, and thrombocytopenia. These were significant findings (*P* value < 0.05—Table 3). Coma and convulsions were the presenting features in 13–18% of admissions. This clinical presentation is in line with other case series [2, 7, 9]. 

Feeding problems often combined with heavy vomiting were found the most common (51–77%) initial symptoms. However, other researchers reported these symptoms slightly more frequent as 83%–91% [2, 7]. The cause may be improvement in management measures and nutrition of PA. Clinical acute or chronic neurological manifestations are caused by accumulation of toxic metabolites [2, 7]. Our 13–68% admissions had CNS symptoms like apathy/lethargy, coma, and seizures. Other reports are also almost similar [2, 7]. Convulsions were reported in 43% of patients [2]. However, the observations in the current study were much lower as 13% only. Similarly, respiratory symptoms like cough, dyspnoea, and apnoea were frequently observed as 36% in our patients, while these figures were reported as 57% in the Austrian study [2]. The difference in frequency of these findings is probably due to prolonged period of the Austrian study (15 years), because complications increase with advancing age of patients with PA. Less common clinical features observed in the present study were acidotic breathing, weight loss, loose motion,, and constipation, in keeping with other reports [2, 12]. 

Regarding physical findings (Table 2), dehydration was found in 92% admissions, which is consistent with other reports [2]. In this study, children showed tendency to failure to thrive that is reflected by their mean weight for age, 71.2% (normal >90%) [10], and mean height/length for age, 91% (normal >95%), Contrarily, some other studies showed more impact of the disease on height, as compared to weight of the patients [2, 13]. This is probably because of advancement in nutrition and management of PA with time, because height is affected considerably in more frequent and prolonged period of decompensations. As shown by Schreiber et al. [1], our patients also revealed some neurological manifestations like encephalopathy, disturbances in tone, and deep tendon reflexes in 5–50% admissions.

Regarding diagnoses of the diseases with which PA patients were admitted, metabolic crises were encountered in 83%, respiratory problems in 30% (odd ratio 1.99—Table 4), and diarrhoea in 9% admissions (Table 2). Although clinical sepsis was suspected in 1/3, admissions, it was proved by blood culture, in 6% cases only, in contrast to three times higher report as 18% [7]. This difference may be due to variable quality of laboratory facilities. 

### 4.2. Laboratory Considerations

For diagnosis of propionic acidaemia, in this study, tandem mass spectrometry results showed mean propionylcarnitine (C3) 18.76 umol/L (normal: <4.33) and mean C3/C2 ratio 1.86 (normal: <0.1). However, Huang and coworkers [14] noted these figures to be a bit lower (12.41 umol/L, and 1.31 umol/L resp.), while another report of C3 level is on a higher side, as 22.13 umol/L [12]. 

In PA, worsening metabolic acidosis has been described in stressful conditions like fever, infection, vomiting, and physical and psychological trauma [9]. In the present study, three-quarter of admissions were found to be associated with metabolic acidosis which is broadly comparable with another report as 90% [2].

In the current study, hyperammonaemia (>80 umol/L) was observed in 96% admissions with mean ammonia level as 308 umol/L and 4.5% admissions showed level >1000 umol/L. Our 6% children, who had hyperammonaemia, underwent peritoneal dialysis for quick recovery. Walter and coresearchers [7] found hyperammonaemia in 100% cases of PA with mean ammonia level as 350 umol/L. Their 18% patients had ammonia level >1000 umol/L who all (100%) underwent peritoneal dialysis. These findings are almost in line with those of the present study. Hypocalcaemia (<8.4 mg/dL) was encountered in 2/3 of admissions (odd ratio 1.82—Table 4) which is comparable with another report [7]. In line with Alberola et al. [5], the current work revealed hypoglycaemia in 12% and hyperglycaemia in 2% admissions. We observed hypocalcaemia (<5.5 g/dL) in 1/4 (one quarter) of admissions as compared to those of the 1/3 [2]. Hypoalbuminemia (<3.5 g/dL) was also noted in three-quarter of admissions in this study. As reported by Delgado and co-researchers [12], the present study also revealed mildly disturbed liver enzymes in 16–40% of admissions. These findings are probably due to impact of metabolic derangements on liver efficiency. Similar to Lehnert and co-workers [2], we noted ketonurea in 1/3 of admissions.

During metabolic crises, propionate inhibits the growth of bone marrow stem cells, leading to leukocytopenia, thrombocytopenia, and/or pancytopenia—a condition which has been described frequently in PA [2]. Neutropenia was reported in 27% and thrombocytopenia in 18% patients [11] as compared to our findings as 36% and 39%, respectively. Anaemia (Hb. <10 g/dL) was found in 26% admissions (odd ratio 0.55—Table 4) as compared to 72% in another report [2]. This difference is probably due to hilly area inhabitants of this study, where comparatively hypoxic environment stimulates erythropoiesis leading to high haemoglobin and also due to advancement in nutrition for PA. Consistently with other reports, radiological studies revealed metabolic bone disease in some of our patients [2, 5]. Similarly, computerized tomography and MRI brain showed generalised brain oedema in 17%, cerebral atrophy in 25%, and multiple cerebral infarcts plus haemorrhage in 2% admissions. These results are consistent with another study [9].

There were some limitations in this study, firstly due to unavailability of complete records of some of the patients, and secondly, this was a retrospective study with little number of patients. Further multicentre, longitudinal studies are needed to better elucidate the clinical spectrum of the disease.

## 5. Conclusion

We have a large gap in knowledge about the path from biochemical abnormality to clinical symptoms and signs of PA. We need to focus our efforts on improving our understanding of PA. This report may be helpful in extending the knowledge about clinical features and disturbances of laboratory parameters in patients with PA. Aggressive identification and initiation of therapy are necessary to prevent severe morbidity and mortality in patients with PA whose life quality is profoundly affected. So, more extensive, centralized newborn screening program is thus imperative.

## Figures and Tables

**Table 1:** Data of 26 patients with PA.

Variable of patients	*n* (%)/mean (SD)	Variables of patients	*n* (%)/mean (SD)
Male	16 (61.5)	No. of admission/patient	5.1 [6.5]
Female	10 (38.5)	Frequency of admission/yr	4.2 [3]
Mean age in yr	2.85 [4.8]	Early onset cases	5.5 [6.7]
Consanguinity of parents	24 (92.3)	Late onset cases	2
H/O of PA in siblings	10 (38.5)	Cerebral palsy	4 (15.4)
H/O NICU admission	17 (63.4)	Seizures	12 (46.2)
No. of deceased patients	2 (7.7)	Hypotonia	11 (42.3)
Time of clinical onset/diagnosis		Hyporeflexia	4 (15.4)
1st week of life (early onset)	22 (84.6)	Hypertonia	4 (15.4)
2nd week—3months (early onset)	2 (7.7)	Hyperreflexia	9 (34.6)
>3 months of age (late onset)	2 (7.7)	*Patients were *	
Diagnosis age (range: 2 day–1yr)	0.13 [0.3]	On NG/G tube feeding	4 (15.4)
Mean % of weight for age	72.4 [17.1]	On home oxygen therapy	2 (7.7)
Mean % of height/length for age	90.7 [8.8]	Look after in social home	2 (7.7)

Values are given in *n* (%) or mean (SD); H/O: history of, NICU: neonatal intensive care unit, NG/G: nasogastric/gastrostomy tube.

**Table 2:** Important clinical, physical, and laboratory findings and diagnoses of diseases on initial presentation in 133 admissions of 26 patients with PA and comparison of these findings with other studies.

Variables	Current study in %	Lehnert et al. 1994 [2]	Other studies	*P* value
*Symptoms *				
Lethargy/apathy/somnolence	68	73	55^‡^	<0.001
Poor feeding and vomiting	77	83	91^‡^	<0.001
Dyspnoea, cough, and others^†^	36	57	—	<0.001
Seizures	13	43	23^¶^	<0.001
Loss of consciousness	18	—	15^¶^	0.013
*Physical findings *				
Muscular hypotonia	62	73	48^¶^	<0.001
Malnutrition	80	59	—	<0.001
*Laboratory Parameters *				
Hyperammonaemia (>80 umol/L)	96	88	100^‡^	0.003
Hypoglycaemia (<50 mg/dL)	12	17	—	<0.001
Hyperglycaemia (>200 mg/dL)	2	17	—	<0.001
Hypoproteinemia (<5.5 g/dL)	27	33	—	0.013
Hypocalcaemia (<8.4 mg/dL)	65	—	36^‡^	<0.001
Pancytopenia	36	41	—	0.013
Anaemia (Hb. < 10 g/dL)	26	72	—	<0.001
Thrombocytopenia (<150,000/mm^3^)	39	62	18^‡^	<0.001
Neutropenia, ANC (<1500 × 10^9^/L)	36	83	27^‡^	0.002
Acidosis pH < 7.35/HCO_3_ < 18 mmol/L	77	90	56^§^	0.245
Alkalosis, pH > 7.45/HCO_3_ > 28 mmol/L	8	—	18^‡^	<0.001
*Diagnosis *				
Sepsis (culture proven)	6	—	18^‡^	<0.001
Suspected sepsis	33	60	85*	<0.001
Death	8	40	32^§^	<0.001

Others^†^: acidotic breathing aspiration, apnoea, suppurative otitis media, sore throat, flu; ^‡^Walter et al., 1995 [7]; ^¶^Schreiber et al., 2012 [1]; ^§^Ozand et al., 1994 [8]; *Chapman et al., 2012 [9].

**Table 3:** Important clinical features and laboratory parameters on initial presentations in *n* = 133 admissions of 26 patients with PA.

Findings/investigations	*n* (%)/mean (SD)	CI, 95%	*P* value
*Symptoms *			
Lethargy	91 (68.4)	85.07–96.93	<0.001
Fever	81 (60.9)	75.72–86.28	<0.001
Poor feeding	68 (51.1)	63.57–72.43	<0.001
Vomiting	102 (76.7)	95.35–108.65	<0.001
Dyspnoea/cough/others^†^	48 (36.1)	44.87–51.13	<0.001
Loss of consciousness	24 (18)	22.44–25.56	0.002
Seizures	17 (12.8)	15.89–18.11	0.028
*Physical findings/diagnosis *			
Dehydration	123 (92.4)	114.99–131.01	<0.001
Hypotonia	81 (61.9)	75.63–86.37	<0.001
Encephalopathy	6 (4.5)	5.61–6.39	0.437
Metabolic crisis	111 (83.4)	103.77–118.23	<0.001
Respiratory diseases^‡^	40 (30.1)	37.39–42.61	<0.001
Coma	6 (4.5)	5.61–6.39	0.437
Sepsis (culture positive)	8 (6)	7.48–8.52	0.300
*Laboratory parameters *			
S. ammonia (umol/L)	308.3 [279.1]	284.10–332.50	<0.001
S. glucose (mg/dL)	91.1 [67.5]	90.54–91.66	0.262
S. total protein (g/dL)	5.75 [1]	5.61–5.77	0.863
S. albumen (g/dL)	2.97 [0.7]	2.91–3.03	0.904
S. calcium total (mg/dL)	8.04 [1]	7.95–8.13	0.863
Haemoglobin (g/dL)	10.96 [2.2]	10.77–11.15	0.703
RBCs count (×10^6^/mm^3^	4.23 [0.9]	4.15–4.31	0.876
Platelets count (×10^3^/mm^3^)	206 [144]	193.51–218.49	<0.001
TLC (×10^3^/mm^3^)	5.34 [3.1]	5.07–5.61	0.592
pH	7.24 [0.21]	7.22–7.26	0.972
Base excess (mmol/L)	−14.6 [6.9]	14.00–15.20	0.234
HCO_3_ (mmol/L)	13.2 [5.95]	12.68–13.72	0.304

^†^Acidotic breathing aspiration, apnoea, suppurative otitis media, sore throat, flu; ^‡^pneumonia, chronic lung disease, upper respiratory tract infection, and hyper reactive airway disease; RBCs: red blood cells, TLC: total leukocyte count, ANC: absolute neutrophil count, HCO_3_: bicarbonate.

**Table 4:** Gender-wise odd ratio analyses for clinical and laboratory findings.

Lethargy/apathy/somnolence	Male	Female	Total
Yes	12	5	17
No	4	5	9
Odds ratio 1.69, chi square 0.234, *P* value 2.485			

Respiratory diseases			
Yes	7	6	13
No	9	4	13
Odds ratio 1.99, chi square 0.65, *P* value 0.428			

Hypocalcaemia (<8.4 mg/dL)			
Yes	9	7	16
No	7	3	10
Odds ratio 1.815, chi square 0 .589, *P* value 0.585			

Anaemia (haemoglobin < 10 g/dL)			
In the range	5	2	7
Outside the range	11	8	19
Odds ratio 0.55, chi square 0.529, *P* value 0.658			

## Abbreviations

PA: Propionic acidaemia
KSA: Kingdom of Saudi Arabia
H/O: History of
NICU: Neonatal intensive care unit
NG: Nasogastric (tube)
ANC: Absolute neutrophil count
C3: Propionylcarnitine
MRI: Magnetic resonance imaging
CNS: Central nervous system.
